# Folate Receptor-Targeting and Reactive Oxygen Species-Responsive Liposomal Formulation of Methotrexate for Treatment of Rheumatoid Arthritis

**DOI:** 10.3390/pharmaceutics11110582

**Published:** 2019-11-06

**Authors:** Minglei Chen, Kambere Amerigos Daddy J.C., Zhigui Su, Nida El Islem Guissi, Yanyu Xiao, Li Zong, Qineng Ping

**Affiliations:** State Key Laboratory of Natural Medicines, Department of Pharmaceutics, China Pharmaceutical University, Nanjing 210009, China; chenminglei_042005@163.com (M.C.); szg707@126.com (Z.S.); setif.nidaa@yahoo.fr (N.E.I.G.); cpuyanyuxiao@163.com (Y.X.)

**Keywords:** rheumatoid arthritis, folate receptor, active targeting, reactive oxygen species-responsive drug release, methotrexate, catalase, oxygen-generation

## Abstract

Multifunctional nanomedicines with active targeting and stimuli-responsive drug release function utilizing pathophysiological features of the disease are regarded as an effective strategy for treatment of rheumatoid arthritis (RA). Under the inflammatory environment of RA, activated macrophages revealed increased expression of folate receptor and elevated intracellular reactive oxygen species (ROS) level. In this study, we successfully conjugated folate to polyethylene glycol 100 monostearate as film-forming material and further prepared methotrexate (MTX) and catalase (CAT) co-encapsulated liposomes, herein, shortened to FOL-MTX&CAT-L, that could actively target to activated macrophages. Thereafter, elevated intracellular hydrogen peroxide, the main source of ROS, diffused into liposomes and encapsulated CAT catalyzed the decomposition of hydrogen peroxide into oxygen and water. Continuous oxygen-generation inside liposomes would eventually disorganize its structure and release the encapsulated MTX. We characterized the in vitro drug release, cellular uptake and cytotoxicity studies as well as in vivo pharmacokinetics, biodistribution, therapeutic efficacy and safety studies of FOL-MTX&CAT-L. In vitro results revealed that FOL-MTX&CAT-L possessed sufficient ROS-sensitive drug release, displayed an improved cellular uptake through folate-mediated endocytosis and exhibited a higher cytotoxic effect on activated RAW264.7 cells. Moreover, in vivo results showed prolonged blood circulation time of PEGylated liposomes, enhanced accumulation of MTX in inflamed joints of collagen-induced arthritis (CIA) mice, reinforced therapeutic efficacy and minimal toxicity toward major organs. These results imply that FOL-MTX&CAT-L may be used as an effective nanomedicine system for RA treatment.

## 1. Introduction

Rheumatoid arthritis (RA) is a chronic inflammatory relapsing autoimmune disorder that causes progressive articular destruction and associated comorbidities in vascular, metabolic, bone and psychological domains [1]. RA has a prevalence of ~1% and affects three times more women than men [2]. It is commonly diagnosed in the middle-aged population (40–60 years of age) [3]. Current treatment strategies for RA include nonsteroidal anti-inflammatory drugs (NSAIDs), glucocorticoids (GCs), disease-modifying anti-rheumatic drugs (DMARDs) and biologic response modifiers (“biologicals”). Typically, methotrexate (MTX), an antimetabolite-folate antagonist of DMARDs family, has gained popularity among rheumatologists in a large range of countries as the first-line antirheumatic agent due to its outstanding effectiveness [4,5,6]. However, long-term treatment or high doses of MTX could cause severe side effects including gastrointestinal disorders, hepatic dysregulations, pneumonitis, hematologic disorders, infections, nephrotoxicity, dermatitis and so forth [7,8,9]. In order to overcome this shortcoming, nanomedicine technologies, the combination of different types of nanocarriers and medicine, were applied to provide multifaceted solutions [10,11]. For RA treatment, an ideal and effective nanomedicine system generally involves—(a) delivery of antirheumatic drugs to the inflamed tissues, cells or subcellular domains using passive and/or active targeting strategy and (b) subsequent rapid drug release from nanocarriers by some specific stimuli-responsive mechanism to reach effective therapeutic concentration within the inflammatory sites.

When RA occurred, abnormal vasculature and impaired lymphatic drainage was observed in inflamed joints representing an enhanced permeability and retention (EPR) effect that is similar to solid tumor. Wide gaps between endothelial cells would allow nanocarriers with appropriate size (ranging from 10 to 200 nm) to penetrate into the synovial tissue by passive targeting [11]. In addition, during the progression of RA, inflammatory cells including macrophages, dendritic cells, T and B lymphocytes and neutrophils infiltrate the RA joints synovium and contribute to the severe destruction of joint and cartilage [12]. In particular, macrophages play a vital role due to their high abundance in inflamed synovial membrane, their activation status and their successful response to anti-rheumatic therapy [13,14,15]. After recruitment, macrophages become “activated macrophages” displaying different phenotypes and then release pro-inflammatory cytokines such as TNF-α and IL-β, chemokines, digestive enzymes, prostaglandins and reactive oxygen species (ROS), which can aggravate or accelerate damage to the normal tissues [16]. Therefore, macrophages have emerged as a potential target for the RA treatment [17]. Several receptors including CD44 [18], CD64 [19], folate receptor-beta (FR-β) [20], vasoactive intestinal peptide receptor [21], scavenger receptor class A [22] and toll-like receptors [23] were over-expressed on activated macrophages, based on which, different types of nanocarriers modified with the corresponding ligands were developed to actively target to activated macrophages [24,25,26,27,28,29]. Among these receptors, FR-β only expressed on activated macrophages involved in inflammatory responses but not on quiescent resident macrophages [30]. In this way, nanomedicine linked with folate could selectively target to pathologic macrophages and relieve the rheumatic symptoms, leaving the healthy macrophages unharmed [30,31]. Nanocarriers such as albumin nanoparticles [27], liposomes [28] and dendrimer [32] modified with folate ligand have been designed and applied to RA treatment.

To date, several stimuli-responsive strategies such as pH-responsive [33], temperature-responsive [34] and enzymatic-cleavable system [35] have been explored for rapid drug release under the pathophysiological environment of RA. In addition, extensive research have found that reactive oxygen species (ROS) comprising hydroxyl radical (OH^−^), hydrogen peroxide (H_2_O_2_), superoxide anion (O_2_^−^) and hypochlorous acid (HOCl) are implicated in the regression of RA [36,37,38,39]. Under the stimulation of inflammatory cytokine such as TNF-α, IL-1β and interferon-γ, ROS was generated by activated macrophages in the synovial membrane, by chondrocytes and also through activated neutrophils in the synovial cavity [36]. This excessive production of ROS can damage protein, lipids, nucleic acids and matrix components in synovial tissue [40], break the balance between oxidant and antioxidant to cause a state of oxidant stress [37] and partially contribute to local tissue hypoxia [41]. In view of this phenomenon, enzymatic antioxidants such as superoxide dismutase (SOD), catalase and glutathione peroxidase as well as non-enzymatic antioxidants including vitamins (A, C and E), β-carotene, antioxidant minerals have been widely used for repairing and maintaining the normal cell redox state of synovium and joints [42]. On the other hand, this abnormally increased ROS level can potentially serve as a stimulus for the design of the ROS-responsive nanomedicine. Representative ROS-responsive nanomaterials include thioether-containing polymers, selenium-containing polymers, tellurium-containing polymers, poly(thioketal) and phenylboronic ester-containing polymers and so forth [43]. However, most of these strategies were used for cancer treatment and few studies associated with RA have been reported [44,45]. Currently, there is an innovative and attractive drug release pattern based on gas generation for example, oxygen-generation and carbon dioxide-generation inside the nanocarriers under specific environments such as thermo-, photo- and ultrasound-conditions and this continuous gas generation could destabilize and destroy the architecture of nanocarriers and eventually promote the drug release [46,47,48,49,50]. Inspired by this, catalase, the well-known natural antioxidant, was incorporated into the PLGA nanoparticles and delivered to the cancer cells [51]. Elevated intracellular level of H_2_O_2_, the major source of ROS, was then penetrated into the nanoparticles and catalyzed by encapsulated catalase through reaction [52] (2H_2_O_2_ → 2H_2_O + O_2_) to generate oxygen, which finally led to the shell rupture of nanoparticles.

**Scheme 1:**
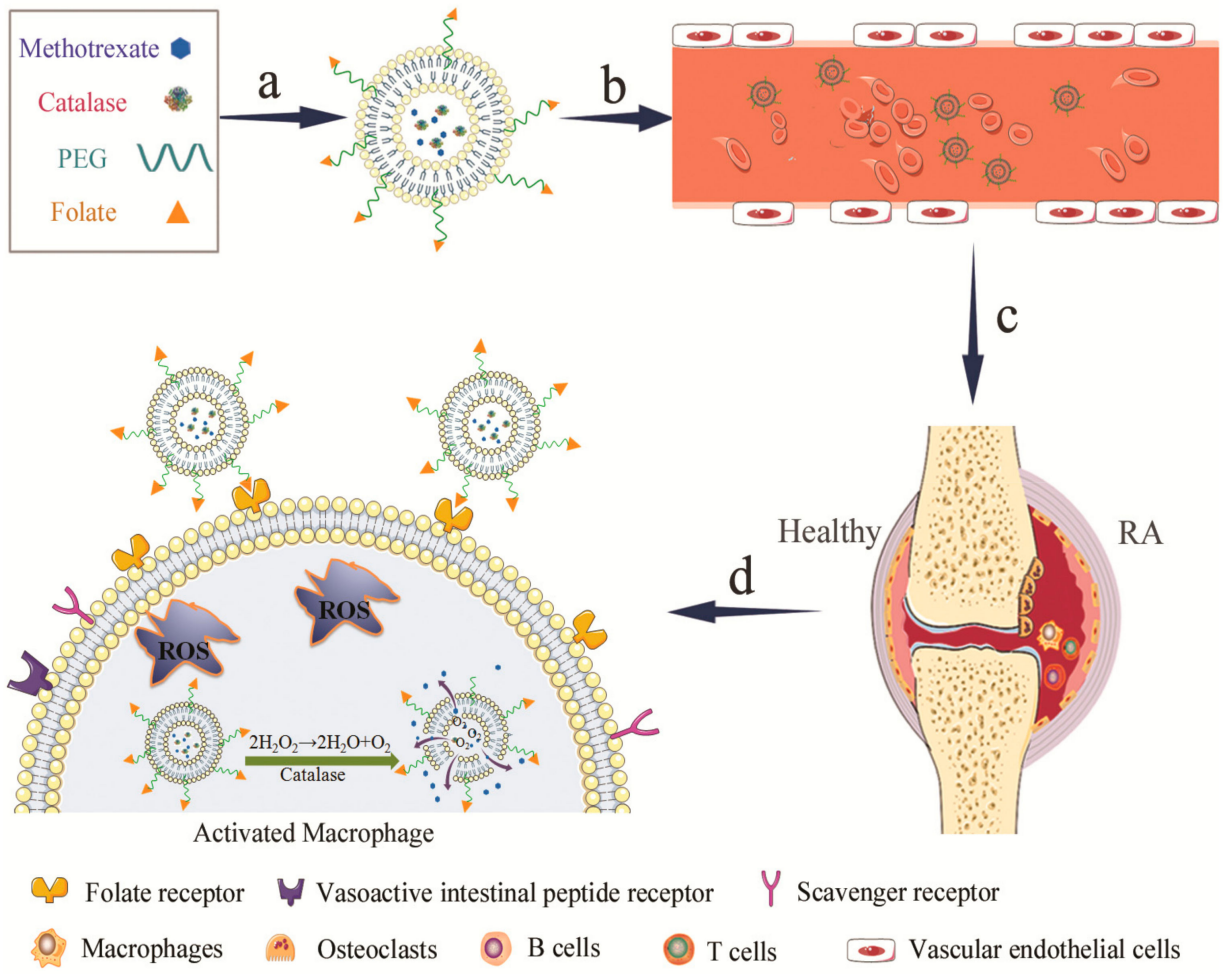
The schematic presentation of liposomes with folate receptor-targeting and reactive oxygen species (ROS)-responsive drug releasing functions for rheumatoid arthritis (RA) treatment. a: Preparation of methotrexate and catalase co-encapsulated liposomes modified with folate-PEG conjugate, among of which, methotraxate was the therapeutic drug, catalase acted as the trigger for ROS-responsive releasing and folate-PEG conjugate played a dual effect for folate receptor targeting and PEGylation of liposomes; b: During the process of blood circulation, PEGylation prevented the liposomes from the phagocytosis of reticuloendothelial system and wide gaps between endothelial cells under pathological conditions facilitate liposomes to penetrate into synovial cavity; c: abundant activated macrophages were distributed over the synovial tissues and were considered as a potential target due to their participation in the progression of RA; d: Activated macrophages represented both increased expression of folate receptor and elevated intracellular ROS level. The former enables the folate receptor-mediated endocytosis of liposomes and the latter promotes the drug release by oxygen-generation (2H_2_O_2_ → 2H_2_O + O_2_ catalyzed by catalase) induced structural destroy of liposomes.

Herein, we design a novel folate receptor-targeting and ROS-responsive liposome co-encapsulating methotrexate and catalase to actively target to activated macrophages through folate receptor-mediated endocytosis and subsequently release the drug by oxygen-generation induced structural failure of liposome through catalyzed reaction between catalase and elevated intracellular H_2_O_2_ for RA treatment. As shown in Scheme 1, we first synthesize folate-PEG (100) monostearate (FOL-S100) as the material for liposomal preparation through the esterification between folate and PEG (100) monostearate. The folate moiety is responsible for folate receptor targeting and the existences of PEG can extend the blood circulation time of liposomes and enhance its penetration into inflamed joints through EPR effects. In addition, liposomes composed of POPC (1-palmitoyl-2-oleoyl-*sn*-glycero-3-phosphocholine) have been developed for encapsulation of enzyme such as alcohol dehydrogenase [53], catalase [54] and glucose oxidase [55]. Based on this approach, methotrexate and catalase were co-encapsulated into liposomes. The former serves as an antirheumatic drug while the latter acts as the trigger for ROS-responsive drug release and also an antioxidant for balancing the redox state. Finally, we evaluated the in vitro physicochemical properties, ROS-responsive drug release, cellular uptake and cytotoxicity as well as in vivo pharmacokinetics, biodistribution, therapeutic efficacy and safety of all prepared liposomes.

## 2. Materials and Methods

### 2.1. Materials

1-Ethyl-3-(3-dimethylamino)propyl carbodiimide hydrochloride (EDC) and *N*-hydroxysuccinimide (NHS) were purchased from Sigma-Aldrich Shanghai Trading Co, Ltd. (Shanghai, China). Poly(ethyleneglycol) (100) monostearate (S100) with PEG molecular weight 4400 g/mol was obtained from Sigma (Milwaukee, WI, USA). Methotrexate hydrate (MTX, purity 99%), doxorubicin (DOX, purity 98%), folate (FOL, purity ≥ 98%), catalase (from bovine liver, ≥3000 units/mg protein), hydrogen peroxide solution (30 wt % in H_2_O), lipopolysaccharides (LPS), Trition X-100 and 1-palmitoyl-2-oleoyl-*sn*-glycero-3-phosphocholine (POPC, purity > 99%) were all purchased from Shanghai Aladdin Co., Ltd. (Shanghai, China). Dimethyl sulfoxide (DMSO), MTT, phosphate-buffered saline (PBS), 4′,6-diamidino-2-phenylindole (DAPI), folate-deficient Dulbecco’s modified eagle mediun (DMEM) medium (Hyclone^®^), penicillin-streptomycin solution, trypsin-EDTA solution (Hyclone^®^) and fetal bovine serum (FBS, Hyclone^®^) were all provided by Sunshine Biotechnology Co., Ltd. (Nanjing, China). Chickens type II collage (2 mg/mL), complete Freund’s adjuvant (CFA, 4 mg/mL) and incomplete Freund’s adjuvant (IFA, 4 mg/mL) were purchased from Chondrex, Inc. (Redmond, WA, USA). All other chemicals and reagents were of HPLC grade.

### 2.2. Cell Cultures

RAW264.7 cells (murine macrophage cells) were obtained from American Type Culture Collection (ATCC). The cells were nurtured in folate-deficient DMEM medium containing 10% (*v*/*v*) FBS, 100 U/mL penicillin and 100 mg/mL streptomycin at 37 °C under an atmosphere of 5% CO_2_ and 90% relative humidity. The medium was renewed every second day until 80% confluence was obtained. Before the experiments, once the precultured cells had reached 75% confluence, the cells were rinsed with PBS and trypsinized before harvest.

### 2.3. Animals

Sprague-Dawley rats and C57BL/6 mice were obtained from the Shanghai Silaike Laboratory Animal Co., Ltd. (Shanghai, China). All the animals were subjected to regular 12 h light-dark cycle and kept in a controlled temperature and humidity standard animal room. All animal experiments were performed in accordance with Provision and General Recommendation of Chinese Experimental Animals Administration Legislation and the Instructive Notions with Respect to Care for Laboratory Animals and were approved by Animal Ethics Committee of China Pharmaceutical University and the Department of Science and Technology of Jiangsu Province (license number—SYXK(SU)2016-0011, 27 January 2016).

### 2.4. Synthesis of FOL-S100

**Figure 1:**
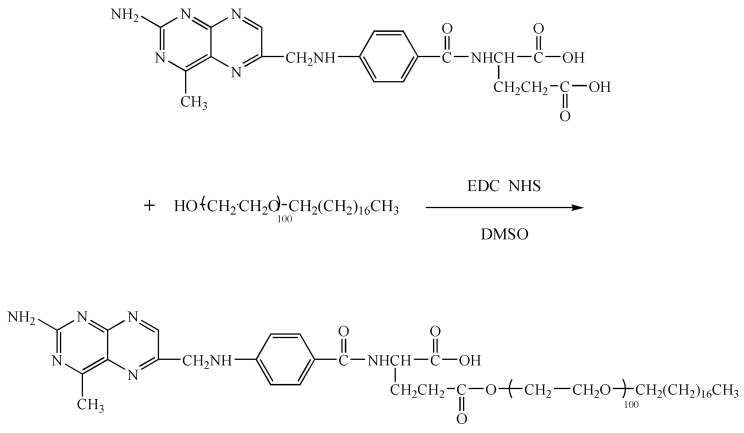
Scheme for synthesis of FOL-S100.

The synthesis of FOL-S100 was performed according to reference [56] (as seen in Figure 1.). In brief, 0.883 g of FOL (2 mmol), 4.684 g of S100 (1 mmol), 0.192 g of EDC (1 mmol) and 0.138 g of NHS (1.2 mmol) were dissolved in 250 mL of dimethyl sulfoxide (DMSO) and stirred at room temperature for 24 h. The resultant solution was placed into a pre-swelled dialysis bag (3.5 kDa MW cutoff, Sigma, USA) and dialyzed in 20 L of NaHCO_3_ buffer (pH 9.0) for 12 h (change the buffer every 3 h) and then transferred to 20 L of deionized water for 24 h (change the deionized water every 4 h) to remove the un-reacted materials and byproducts. Thereafter, lyophilization procedure was applied to remove water and trace amounts of organic solvent. The synthetic product was further purified by column chromatography on silica gel H by using chloroform/methanol (12:1, *v*/*v*) as the eluent. Finally, the structure of purified product was confirmed by ^1^H nuclear magnetic resonance (NMR) (300 MHz, D_2_O) and Fourier transform infrared (FT-IR) (KBr pellet) spectroscopy. Considering that a maximal UV absorbance at 285 nm was observed to determine the concentration of conjugated FOL [57], a known amount of dried FOL-S100 was dissolved in DMSO and then the conjugation efficiency of FOL to S100 was calculated by measuring the UV absorbance value of FOL-S100 at 285 nm. Serially diluted concentrations of FOL in DMSO were used to construct a calibration curve.

### 2.5. Preparation of FOL-MTX&CAT Liposomes

Liposomes were prepared on the basis of the method used by Yoshimoto M. et al. [54]. In a round bottom flask, 200 mg of POPC, 14.8 mg of cholesterol and 60 mg of FOL-S100 were dissolved in 10 mL of methanol/dichloromethane mixture (1:1, *v*/*v*). The organic solvent was evaporated under vacuum at 25 °C to form the lipid film and then the flask was transferred to the desiccators under vacuum overnight to completely remove the residual solvent. The lipid film was hydrated with 5 mL PBS (0.1 mM, pH 8.0) containing MTX and CAT at the concentrations of 5 mg/mL and 2 mg/mL, respectively. The multilamellar vesicles (MLVs) formed was frozen at −80 °C and thawed at 25 °C to enhance the transformation of small liposomes to larger ones. This freezing and thawing treatment was performed eight times. The final MLVs were extruded through a LiposoFast^TM^-basic extruder (Avestin, Ottawa, Canada) using polycarbonate membranes with nominal pore diameters of 200 nm and 100 nm. Extrusion through 200 nm membranes preceded extrusion with 100 nm membranes and extrusion through each polycarbonate membrane was performed fifteen times to obtain the MTX and CAT co-encapsulated liposome decorated with folate ligand, which was donated as FOL-MTX&CAT-L Additionally, we prepared two more liposomes as comparisons by using the exact same method with slight modification. One liposome was prepared by using 60 mg of S100 instead of FOL-S100 and hydration solution only contained 5 mg/mL of MTX while another liposome was constructed by using 60 mg of S100 instead of FOL-S100 and hydration solution contained 5 mg/mL of MTX and 2 mg/mL of CAT, which were referred as MTX-L and MTX&CAT-L, respectively.

### 2.6. Characterization of Liposomes

Particle size, zeta potential and size distribution of MTX-L, MTX&CAT-L and FOL-MTX&CAT-L were assessed by dynamic light scatting (DLS, 90 Plus Particle Size, Brookhaven Instruments Co. NY, USA). In addition, the morphological observations were performed under transmission electron microscopy (TEM, H-600, Hitachi, Tokyo, Japan) following negative staining with 0.1 wt % sodium phosphotungstate solution.

The free MTX and CAT were separated from the liposomes by ultracentrifugation and gel column chromatography, respectively. Specifically, the liposomes containing free MTX were transferred to an ultracentrifugal filter device (3kDa MWCO, Millipore, Bedford, MA, USA) and centrifugated at 12,000 rpm for 20 min. Supernatant free MTX was collected and further quantitatively determined by Agilent 1100 HPLC system (Agilent, Palo Alto, CA, USA) equipped with YMC ODS column (250 × 4.6 mm, 5 μm). The column was kept at 25 °C and the mobile phase consisted of acetonitrile and potassium phosphate (12:88, *v*/*v*, pH 4.5) with a flow rate of 1.0 mL/min. The injection volume was 20 μL and the effluent was monitored by UV detector at 302 nm. As for the separation of CAT, 0.5 mL of liposomes containing free CAT were added into a 15 cm Sepharose 4B gel column with PBS solution (pH 7.0) as the eluent. After the collection of liposomes, 1% triton X-100 was added as the demulsifier and the amount of CAT was determined according to BCA Protein Assay Kit (Beyotime Biotec Co. Ltd., Shanghai, China). The encapsulation efficiency (EE) and drug loading (DL) of MTX and CAT were calculated by using the following Equations (1–4):

(1) $${\mathrm{EE}}_{\mathrm{MTX}}=\frac{{\mathrm{Weight}\;\mathrm{of}\;\mathrm{Total}}_{\mathrm{MTX}}-{\mathrm{Weight}\;\mathrm{of}\;\mathrm{Free}}_{\mathrm{MTX}}}{{\mathrm{Weight}\;\mathrm{of}\;\mathrm{Total}\;}_{\mathrm{MTX}}}\times 100\%$$

(2) $${\mathrm{DL}}_{\mathrm{MTX}}=\frac{{\mathrm{Weight}\;\mathrm{of}\;\mathrm{Total}}_{\mathrm{MTX}}-{\mathrm{Weight}\;\mathrm{of}\;\mathrm{Free}}_{\mathrm{MTX}}}{\mathrm{Weight}\;\mathrm{of}\;\mathrm{Total}\;\mathrm{liposome}}\times 100\%$$

(3) $${\mathrm{EE}}_{\mathrm{CAT}}=\frac{{\mathrm{Weight}\;\mathrm{of}\;\mathrm{Encapsulated}}_{\mathrm{CAT}}}{{\mathrm{Weight}\;\mathrm{of}\;\mathrm{Total}\;}_{\mathrm{CAT}}}\times 100\%$$

(4) $${\mathrm{DL}}_{\mathrm{CAT}}=\frac{{\mathrm{Weight}\;\mathrm{of}\;\mathrm{Encapsulated}}_{\mathrm{CAT}}}{\mathrm{Weight}\;\mathrm{of}\;\mathrm{Total}\;\mathrm{liposome}}\times 100\%$$

To investigate the influence of liposomal preparation on the activity of CAT, the activity of CAT before and after liposomal encapsulation was determined. In brief, demulsifier, that is, 1% triton X-100 was added to FOL-MTX&CAT-L to destroy the formulation and release the encapsulated CAT. The activity of encapsulated CAT was subsequently assessed by Catalase Assay Kit (Beyotime Biotec Co. Ltd., China) according to the manufacturer’s instructions [58]. Similarly, activity of free CAT was determined using the same method as a comparison. In addition, the amount of CAT was also measured by BCA Protein Assay Kit (Beyotime Biotec Co. Ltd., China) to calculate the specific activity of CAT (U/mg). It should be noted that other compositions of liposomes such as POPC and cholesterol as well as demulsifier had negligible effects on the CAT activity measurements.

### 2.7. In Vitro Drug Release

In vitro drug release of MTX-L, MTX&CAT-L and FOL-MTX&CAT-L were performed by a membrane dialysis technique. In order to investigate ROS-responsive drug release capability of CAT encapsulated liposomes, 1 mL of MTX-L, MTX&CAT-L and FOL-MTX&CAT-L was placed into a dialysis bag (MWCO of 8–12 kDa) and the dialysis bag was tightened and soaked in 50 mL of 0.9% saline with or without 1 mM H_2_O_2_. The experiments were carried out at 37 °C and stirred at 100 rpm in three replicates. At predetermined intervals, 0.5 mL of samples were withdrawn and replaced with equal amount of pre-warmed fresh medium. The concentration of MTX in different samples was analyzed by HPLC. Furthermore, the dynamic amount of H_2_O_2_ remained in medium was also determined by Hydrogen Peroxide Assay Kit (Beyotime Biotec Co. Ltd., China) using the method of ferrous oxidation in xylenol orange (FOX) assay [59].

To further investigate the influence of concentration of H_2_O_2_ on drug release, different amount of H_2_O_2_ was added into 0.9% saline to achieve different final concentrations of H_2_O_2_ (0, 0.05, 0.1, 0.5, 1 and 2 mM). The release behaviors of MTX in MTX-L, MTX&CAT-L and FOL-MTX&CAT-L were analyzed by the same above-mentioned procedure. In addition, aliquots of FOL-MTX&CAT-L were added into 0.9% saline with or without 1 mM of H_2_O_2_ and incubated for 1, 4 and 8 h, respectively. The dynamic morphology of FOL-MTX&CAT-L under different conditions was observed by TEM (H-600, Hitachi, Japan).

### 2.8. Validation of LPS Activated RAW264.7 Cell Model

Previous research has reported that RAW 264.7 cells activated by lipopolysaccharide (LPS) could spontaneously reveal increased intracellular ROS level and increased expression of FR over the cell surface [60]. Inspiring by this result, in our study, RAW 264.7 cells were incubated with different concentrations of LPS for different incubation time and then intracellular ROS level and expression of FR were further assessed to choose the proper activation condition. Specifically, RAW 264.7 cells were seeded in 6-well plate at a density of 5 × 10^5^ cells per well and incubated with 50, 100, 500 and 1000 ng/mL concentration of LPS for 6, 12, 24 and 48 h, respectively. Quantitative intracellular ROS generation was detected using 2′,7′-Dichlorodihydrofluorescein diacetate (DCFH-DA) Reactive Oxygen Species Assay Kit (Beyotime Biotec Co. Ltd., China) [61]. At the end of incubation period, the supernatant was removed and the wells were washed three times with ice-cold PBS. After treatment with DCFH-DA for 20 min at 37 °C, the cells were washed three times with ice-cold PBS, harvested and resuspended in PBS. The fluorescence was measured by flow cytometry (FACS-Calibur, BD Biosciences). Meanwhile, the fluorescence of RAW 264.7 cells without LPS activation was measured as negative control.

The expression of folate receptor (FR) over the surface of RAW 264.7 cells before and after LPS activation were evaluated by an indirect confocal laser scanning microscope (CLSM) method. We first chose doxorubicin (DOX) as the model drug due to its fluorescent property and then prepared the corresponding liposomes by replacing MTX with DOX using the same processing method with slight modification that 500 μg of DOX was dissolved with POPC, cholesterol and S100 (or FOL-S100) in organic mixture to form the lipid film and the remaining procedures kept the same. The final prepared liposomes were referred to as DOX-L, DOX&CAT-L and FOL-DOX&CAT-L, respectively. RAW 264.7 cells were seeded in 6-well plate at a density of 5 × 10^5^ cells per well and incubated with 1000 ng/mL of LPS for 48 h at 37 °C, which could cause the highest intracellular ROS level.The medium was then replaced with fresh culture medium containing DOX&CAT-L or FOL-DOX&CAT-L, respectively and incubated for 4 h at 37 °C. Cells were then rinsed three times with ice-cold PBS, fixed with 4% paraformaldehyde and stained with 4′,6-diamidino-2-phenylindole (DAPI) nuclei dye. Lastly, the cellular uptake of DOX&CAT-L and FOL-DOX&CAT-L were observed using confocal laser scanning microscope (CLSM) (ZEISS LSM 700 SYSTEM, Oberkochen, Germany). RAW 264.7 cells without LPS activation were used as negative control.

### 2.9. Intracellular Uptake of Liposomes

RAW 264.7 cells were seeded in 24-well plate at a density of 1 × 10^5^ cells per well and incubated with or without 1000 ng/mL of LPS for 48 h at 37 °C. To investigate time-dependent uptake, the medium was replaced with DOX-L, DOX&CAT-L and FOL-DOX&CAT-L at a DOX concentration of 10 μg/mL and incubated for 1, 2, 4 and 8 h at 37 °C. To investigate concentration-dependent uptake, the medium was replaced with DOX-L, DOX&CAT-L and FOL-DOX&CAT-L at a DOX concentration of 1, 5, 10 and 20 μg/mL and incubated for 8 h at 37 °C. At the end of the incubation period, all the wells were washed three times with ice-cold PBS and the cells were subsequently incubated with 150 μL of cell lysis buffer for 10 min at 37 °C. The samples were centrifuged at 12,000 rpm for 10 min to remove the cellular debris. 20 μL aliquot of each cell lysate was collected to determine the cell protein content using BCA Protein Assay Kit (Beyotime Biotec Co. Ltd., China). The concentration of DOX in cell lysate was measured by Shimadzu 10A vp HPLC system (Kyoto, Japan) equipped with a YMC ODS column (250 × 4.6 mm, 5 μm). The column was kept at 35 °C and the mobile phase consisted of methanol: ammonium acetate (50: 50, *v*/*v*, pH 3.6) adjusted with acetic acid. The flow rate was 1.0 mL/min. The injection volume was 20 μL and effluent was monitored by a fluorescence detector (Shimadzu, Kyoto, Japan) with excitation wavelength of 495 nm and emission wavelength of 560 nm. The uptake index (UI) was calculated by using the Equation (5) [62]:

(5) UI=C/P

where C is the concentration of DOX in cell lysate and P is the concentration of protein in cell lysate.

### 2.10. In Vitro Cytotoxicity Study

The cytotoxicity of MTX solution (MTX-S), MTX-L, MTX&CAT-L and FOL-MTX&CAT-L was evaluated by determining the cell viability using MTT assays. RAW 264.7 cells were seeded in 96-well plate at a density of 1 × 10^4^ cells per well and incubated with 1000 ng/mL of LPS for 48 h at 37 °C. The medium was then substituted with MTX-S, MTX-L, MTX&CAT-L and FOL-MTX&CAT-L containing the same concentrations of MTX (0.01–20 μg/mL) diluted in folate-deficient DMEM and incubated for 24 h at 37 °C. After incubation, 10 μL of MTT (5 mg/mL) was added and incubated for another 4 h. The medium was replaced with 150 μL of DMSO to solubilize the formazan crystals. The UV absorbance intensity of cells was measured by Microplate Reader (Thermo Electron Corporation, Waltham, MA, USA) at 570 nm. Each point was performed in triplicates. The viability percentage was assessed using the following Equation (6) [63]:

(6) $$\mathrm{Cell}\;\mathrm{viability}\;(\%)=\frac{{\mathrm{OD}}_{S}-{\mathrm{OD}}_{N}\;}{{\mathrm{OD}}_{P}-{\mathrm{OD}}_{N}}\times 100\%$$

where OD_S_ was the absorbance of each sample well, OD_N_ was the absorbance of negative-control wells (PBS) and OD_P_ was the absorbance of positive-control wells (medium).

### 2.11. Pharmacokinetic and In Vivo Biodistribution Studies

To explore the pharmacokinetics of MTX-S, MTX-L, MTX&CAT-L and FOL-MTX&CAT-L, male Sprague Dawley rats weighing 200–220 g were randomly divided into four groups (*n* = 6) and then MTX-S, MTX-L, MTX&CAT-L and FOL-MTX&CAT-L were administered intravenously at an MTX dose equivalent to 4 mg/kg. Blood collections were performed by retro-orbital puncture with the aid of a glass capillary at 5, 15, 30, 60, 120, 240, 480, 680, 720 and 1440 min after intravenous administration. All blood samples were collected in heparinized tubes and subjected to centrifugation at 3000 rpm for 10 min. Plasma was carefully collected and stored at −20 °C for subsequent analysis. The concentrations of MTX in the blood were detected by HPLC and the pharmacokinetic parameters were analyzed by Kinetica 4.4 (Thermo Electron Corporation, Waltham, MA, USA).

In vivo biodistribution of MTX-S, MTX-L, MTX&CAT-L and FOL-MTX&CAT-L were evaluated in collage induced arthritis (CIA) mice model, which was established as previously described [64]. To induce CIA, C57BL/6 mice were injected intradermally at the base of the tail with 100 μL of emulsion containing chicken type II collagen (2 mg/mL, Chondrex, Redmond, WA, USA) and Complete Freund’s Adjuvant (CFA, 4 mg/mL, Chondrex, USA) as the initial immunization. After 21 days, mice were subjected to a boost immunization with 100 μL of emulsion containing chicken type II collagen and Incomplete Freund’s Adjuvant (IFA, 4 mg/mL, Chondrex, USA) at the same concentration. After 41 days, CIA mice were received a single intravenous administration of MTX-S, MTX-L, MTX&CAT-L and FOL-MTX&CAT-L via tail vein at a dose of 4 mg/kg (*n* = 3). The organs of interest were collected at 0.5, 2, 4, 8, 12, 24 h after administration. The samples were rinse in saline, blotter dry, weighed and then frozen at −20 °C until assay. The concentrations (expressed as μg MTX/g organ) of MTX in homogenized tissues were determined by HPLC.

Furthermore, in vivo fluorescence imaging experiments were performed to confirm the biodistribution of MTX-L, MTX&CAT-L and FOL-MTX&CAT-L. DiR(1,1-dioctadecyl-3,3,3,3-tetramethylindotricarbocyanine iodide), a hydrophobic near infrared dye, was loaded into three liposomes, respectively. CIA mice were intravenously administrated with DiR loaded liposomes (2.5 mg/kg) and anesthetized using intraperitoneal injection of chloral hydrate (10 mg/kg). In vivo imaging tests were subsequently performed by collecting pictures within the fixed excitation wavelength of 720 nm and emission wavelength of 790 nm at different time points (2, 4, 12 and 24 h).

### 2.12. Therapeutic Efficacy of Liposomes in CIA Mice

CIA mice were randomly divided into five groups with six mice in each group. On the day of the booster immunization (day 21), CIA mice were intravenously injected with saline, MTX-S, MTX-L, MTX&CAT-L and FOL-MTX&CAT-L at a MTX dose of 1 mg/kg and thereafter once every other day for a total of five injections (day 25, 29, 33, 37 and 41, respectively). Healthy C57BL/6 mice were maintained in parallel as the control group. Arthritis index (AI) [64] and paw thickness [65] of each CIA mice were measured and recorded. Of note, the AI value and paw thickness was measured in a blinded manner. In order to maintain the consistency, one researcher performed all the measurements.

At the end of observation period, mice of each group were sacrificed. Blood was collected and centrifuged at 3000 rpm for 10 min to obtain the serum samples. Pro-inflammatory cytokines including TNF-α and IL-1β were measured by using enzyme linked immunosorbent assay (ELISA) test as per the manufacturer’s guidelines (Beyotime Biotec Co. Ltd., China). Furthermore, total antioxidant capacity of serum in each group was measured using Ferric Reducing Ability of Plasma (FRAP) Assay Kit [66] (Beyotime Biotec Co. Ltd., China).

Finally, to investigate the potential adverse effect of liposomes, body weight of CIA mice in each group was monitored on day 21, 25, 29, 33, 37 and 41, respectively. Additionally, primary organs including heart, liver, spleen, lung and kidney of healthy mice and CIA mice in FOL-MTX&CAT-L group were surgically removed, embedded in paraffin, cut into sections 7 μm thick and stained with hematoxylin and eosin (H&E) for histological evaluation.

### 2.13. Statistical Analysis

Results were expresses as Mean ± S.D. from triplicate measurements, unless otherwise noted. Differences between groups were assessed by two-tailed Student’s *t*-test or one-way ANOVA. Statistical significance was determined at the following thresholds—* *P* < 0.05, ** *P* < 0.01 and *** *P* < 0.001.

## 3. Results and Discussion

### 3.1. Characterization of FOL-S100

**Figure 2:**
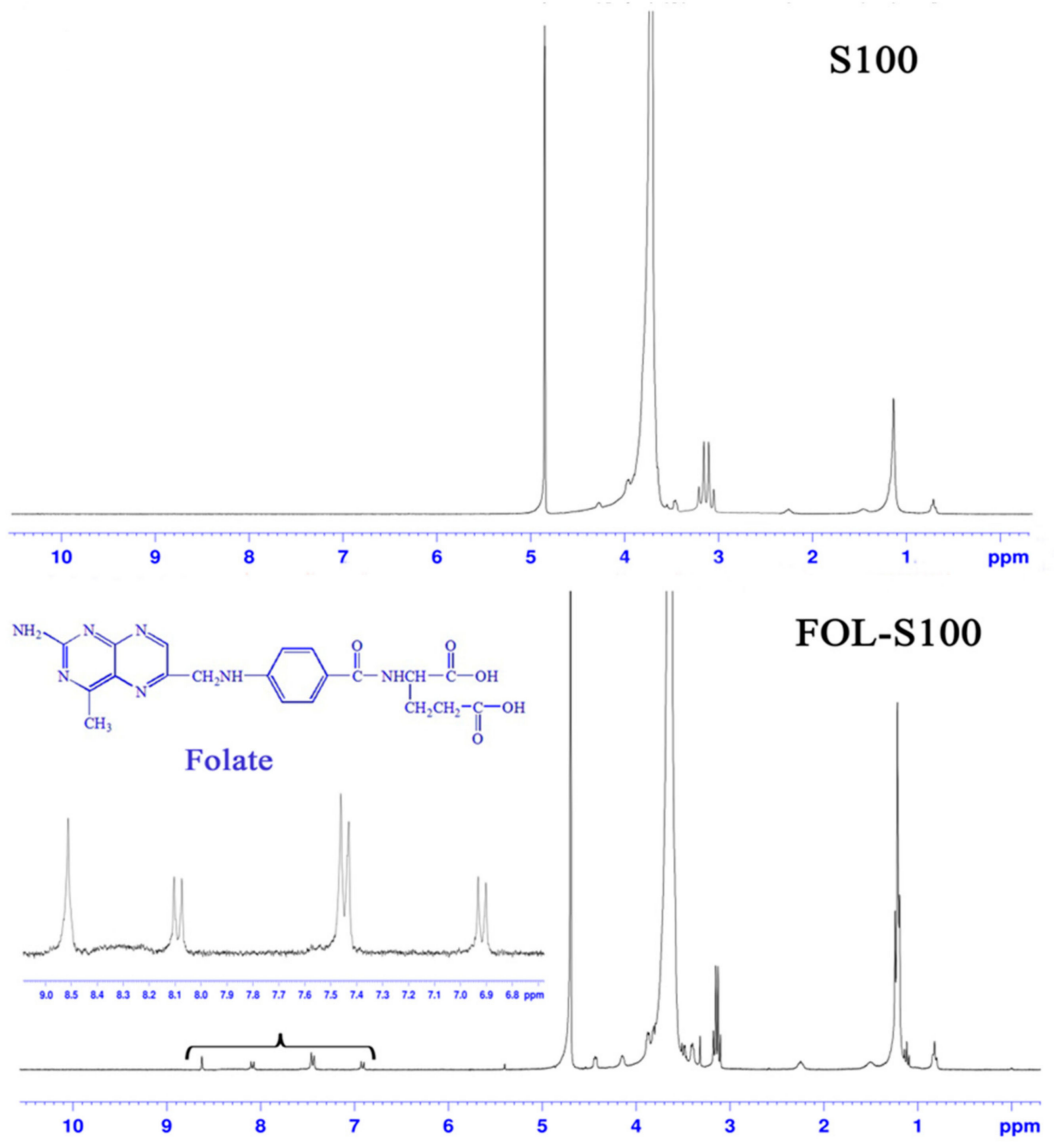
^1^H nuclear magnetic resonance (NMR) spectra of S100 and FOL-S100.

Figure 2 displayed the ^1^H NMR spectra of S100 and FOL-S100. The hydrogen signals at 0.50–1.55 ppm and 3.0–4.2 ppm were assigned to the aliphatic hydrocarbons and PEG of S100, respectively. ^1^H NMR spectra of FOL-S100 showed characteristic signals of FA at 4.47 ppm (methylene proton), 6.91–6.94 ppm and 7.44–7.48 ppm (aromatic protons), 8.1 ppm (aliphatic amide protons) and 8.60 ppm (pteridine proton), which were in accordance with literatures [67,68].

**Figure 3:**
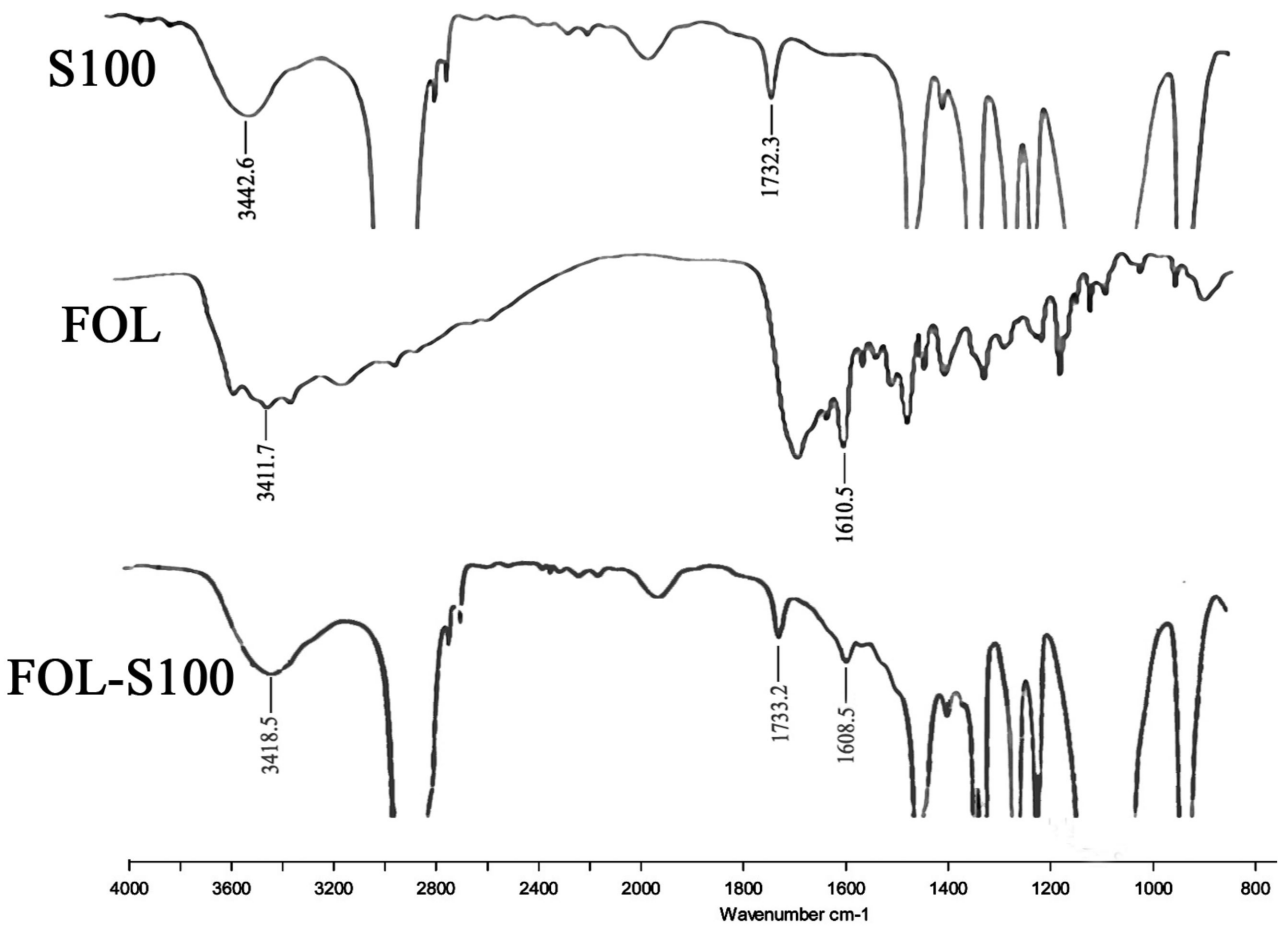
Fourier transform infrared (FT-IR) spectra of S100, FOL and FOL-S100.

The FT-IR spectra of S100, FOL and FOL-S100 were shown in Figure 3. FT-IR spectra of S100 showed free hydroxyl stretching bands at 3442.6 cm^−1^ *ν* (O–H) whereas FOL and FOL-S100 showed free hydroxyl stretching bands at 3411.7 cm^−1^ and 3418.5 cm^−1^ *ν* (O–H), respectively, with a shifting around 20 cm^−1^ towards lower wave band. In addition, 1732.3 cm^−1^ and 1733.2 cm^−1^ *ν* (C=O) of ester linkage in S100 and FOL-S100 were observed. The benzene ring stretching bands of FOL and FOL-S100 located at 1610.5 cm^−1^ and 1608.5 cm^−1^, respectively. Combined with the information of ^1^H NMR, we confirmed that FOL-S100 was successfully synthesized by esterification between the hydroxyl group of S100 and the carboxylic group of FOL. The conjugation efficiency of FOL to S100 was calculated as 85.4% on a molar ratio.

### 3.2. Preparation and Characterization of Liposomes

For the preparation of liposomes, pivotal parameters such as pH of buffer, ionic strength, drug-lipid ratio, freezing–thawing times and extrusion times were investigated to achieve the optimal preparation process (data not shown). We found that pH of buffer significantly influence the encapsulation efficiency of both MTX and CAT. When the pH of buffer was set to 8.0, acidic MTX could be steadily encapsulated in the inner water phase as an ionized state and meantime CAT revealed negative charge under the pH 8.0 of buffer facilitating the electrostatic interaction between the negative charged CAT and electroneutral POPC lipid, which was in accordance with previous research [69,70]. In addition, the encapsulation efficiency of MTX was also associated with dosage of the lipid and drug-lipid ratio while the encapsulation yield of CAT was also influenced by ironic strength and drug-lipid ratio. Based on the optimized formulation processing, relative high encapsulation efficiency and drug loading of both MTX and CAT were obtained.

**Figure 4:**
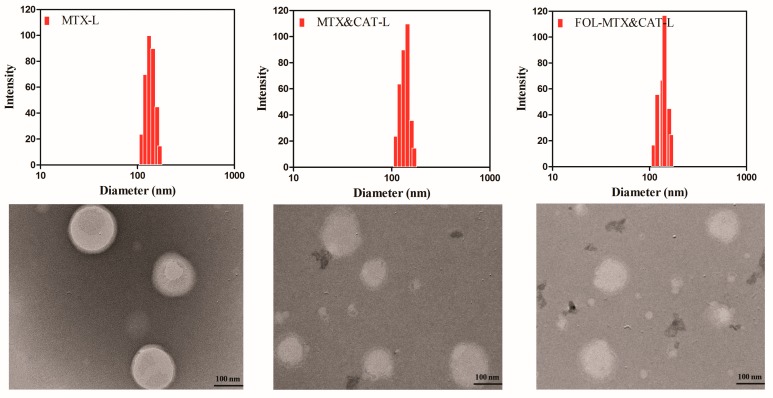
Particle size and transmission electron microscope (TEM) images of MTX-L, MTX&CAT-L and FOL-MTX&CAT-L.

Particle size and zeta potential of all prepared liposomes were shown in Table 1. The mean sizes of MTX-L, MTX&CAT-L and FOL-MTX&CAT-L were 134.9 ± 2.6, 141.2 ± 3.2 and 145.5 ± 4.5 nm, with a zeta potential of −8.5 ± 0.9, −8.8 ± 1.1 and −4.2 ± 0.8 mV, respectively. As mentioned above, inflammatory synovium in RA exhibited abnormal vasculature similar to the phenotypes of solid tumors and wide gaps were formed among the inter-endothelial cell junctions at the inflammatory sites [11]. Therefore, all three liposomes prepared in this study were in the suitable size range that would allow a much-increased accumulation of formulations in the inflammatory tissues through EPR effect. In terms of particle charge, all prepared liposomes showed slightly negative zeta potential owning to the contribution of zwitterionic POPC lipid [71,72,73]. The transmission electron microscopy (TEM) images of all liposomes revealed spherical shapes with homogeneous particle sizes, which were in accordance with PDI results obtained by DLS (Figure 4).

### 3.3. In Vitro Drug Release

**Figure 5:**
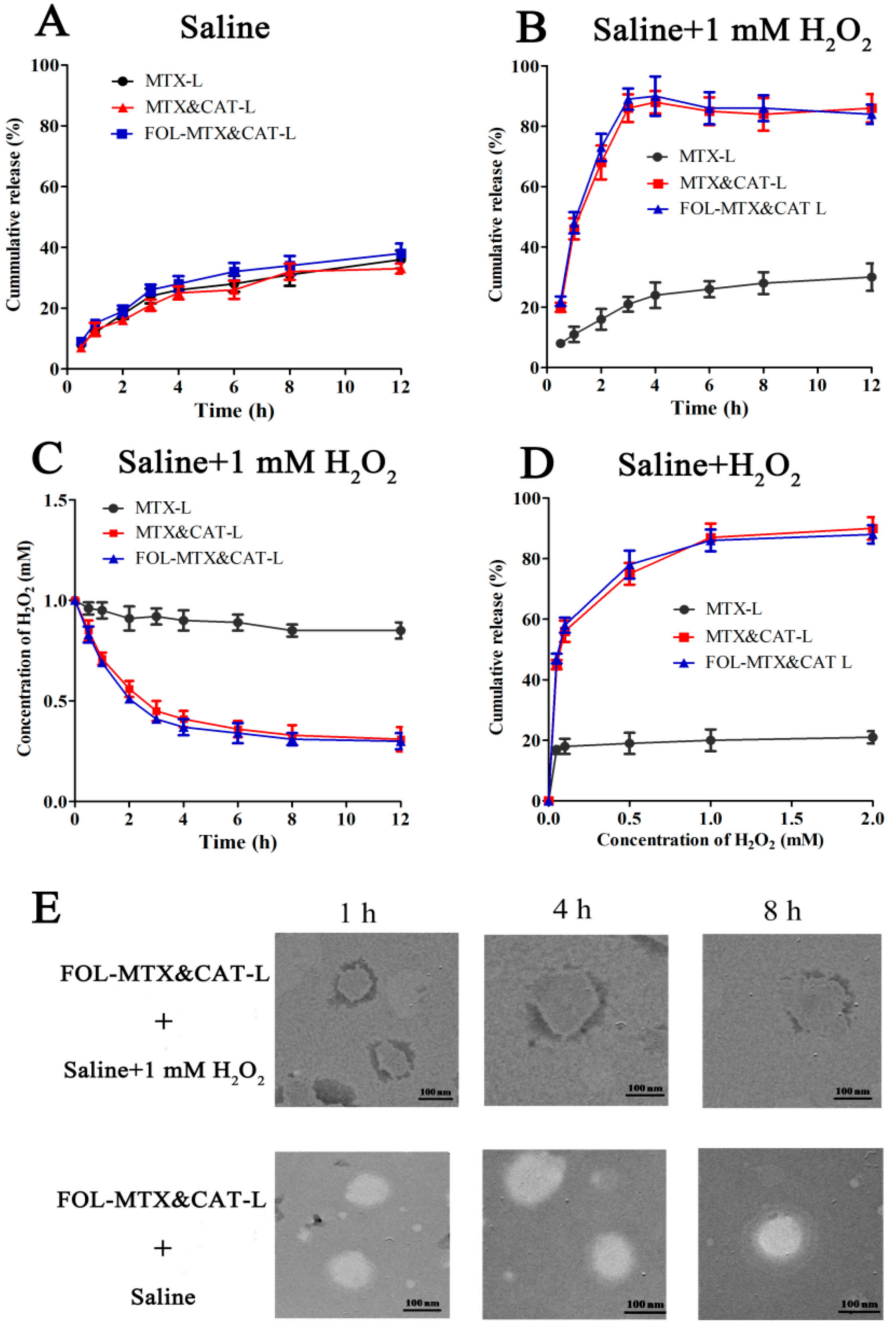
ROS-triggered MTX release from MTX-L, MTX&CAT-L and FOL-MTX&CAT-L (mean ± S.D., *n* = 3). (**A**) The release of MTX at 37 °C from MTX-L, MTX&CAT-L and FOL-MTX&CAT-L in saline. (**B**) The release of MTX at 37 °C from MTX-L, MTX&CAT-L and FOL-MTX&CAT-L in saline with 1mM H_2_O_2_. (**C**) The change of H_2_O_2_ concentration in saline with 1mM H_2_O_2_. (**D**) The release of MTX at 37 °C from MTX-L, MTX&CAT-L and FOL-MTX&CAT-L in saline with different concentrations of H_2_O_2_. (**E**) The TEM images of FOL-MTX&CAT-L in saline with or without 1mM H_2_O_2_ for 1, 4 and 8 h, respectively.

As demonstrated in Figure 5A, all three liposomes exhibited a sustained MTX release in saline. However, the cumulative drug release percentage for MTX&CAT-L and FOL-MTX&CAT-L increased notably in the presence of 1 mM H_2_O_2_. After 3 h, MTX released from MTX&CAT-L and FOL-MTX&CAT-L reached 86.6 ± 3.2% and 89.4 ± 2.6%, respectively. Meanwhile, MTX released from MTX-L was, as expected, barely 21.4 ± 1.8% (Figure 5B). In addition, dynamic concentration of H_2_O_2_ in saline revealed a fast decline in the case of MTX&CAT-L and FOL-MTX&CAT-L. Similarly, negligible consumption of H_2_O_2_ was observed for MTX-L (Figure 5C). Taken together, the burst drug release of MTX&CAT-L and FOL-MTX&CAT-L could be attributed to the collapse of carrier structure caused by the persistent oxygen-generating reaction between encapsulated CAT and H_2_O_2_ in saline. Furthermore, we also investigated the influence of the concentration of H_2_O_2_ upon the drug release. As shown in Figure 5D, higher concentration of H_2_O_2_ in saline led to higher cumulative release of MTX in both MTX&CAT-L and FOL-MTX&CAT-L while negligible influence on MTX-L was observed. Specifically, the cumulative release of MTX in MTX&CAT-L and FOL-MTX&CAT-L reached 56.7 ± 2.5% and 58.2 ± 3.7% in the presence of 0.1 mM H_2_O_2_ while 75.6 ± 4.2% and 78.5 ± 2.6% in the presence of 0.5 mM H_2_O_2_, respectively.

Figure 5E displayed the dynamic morphological changes of FOL-MTX&CAT-L incubated with and without 1 mM H_2_O_2_ by TEM. Upon exposure to 1 mM H_2_O_2_ for 1 h, small pores were observed on the surface of FOL-MTX&CAT-L and the pore size was expanded along with the incubation time. After 4 h, the structure of FOL-MTX&CAT-L was partially broken and finally ruptured after 8 h. In contrast, FOL-MTX&CAT-L without H_2_O_2_ incubation still exhibited spherical and intact morphology, which was consistent with aforementioned drug release behavior of three liposomes.

### 3.4. Intracellular Uptake of Liposomes in LPS Activated RAW 264.7 Cells

**Figure 6:**
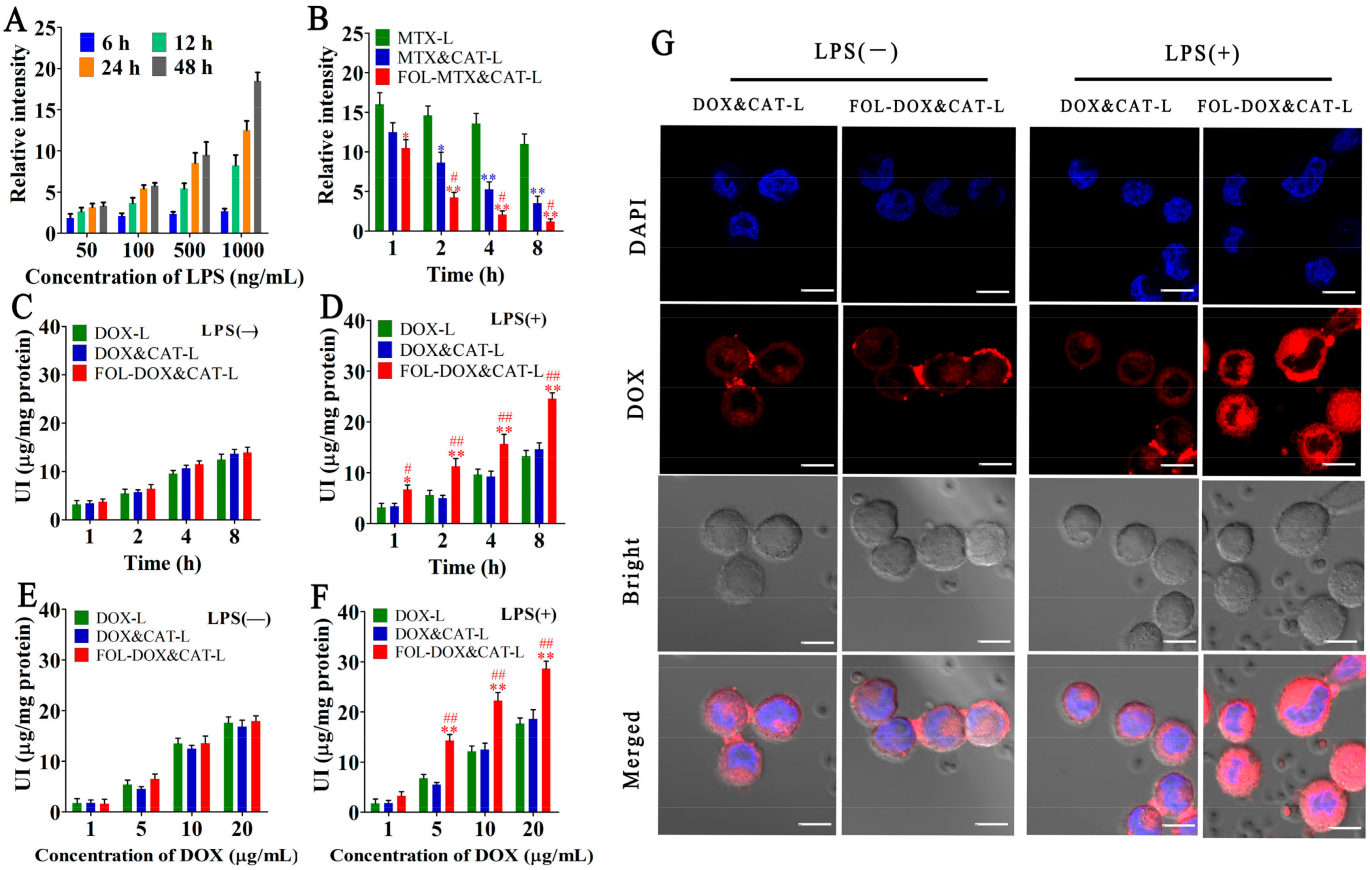
Intracellular uptake of lipsomes in LPS-activated RAW264.7 cells. (**A**) Intracellular ROS levels of RAW264.7 cells after activation by different concentrations of lipopolysaccharide (LPS) for different time period. (**B**) Intracellular ROS levels of LPS-activated RAW264.7 cells after incubation with three liposomes. (**C**–**F**) Time-dependant and concentration-dependant cellular uptake of three liposomes in LPS-activated RAW264.7 cells and untreated RAW264.7 cells, respectively. (**G**) Cellular uptake of DOX&CAT-L and FOL-DOX&CAT-L in LPS-activated RAW264.7 cells and untreated RAW264.7 cells after 4 h incubation observed by confocal laser scanning microscope (CLSM) (blue fluorescence referred to 4′,6-diamidino-2-phenylindole (DAPI) and red fluorescence referred to DOX). Scale bar: 10 μm.

First, we investigated the influence of concentrations of LPS and incubation time on the intracellular ROS levels and expression of FR over the RAW 264.7 cell. As shown in Figure 6A, intracellular ROS levels of RAW 264.7 cells increased under activation with LPS by concentration-dependent and time-dependent pattern. After incubation with 1000 ng/mL of LPS for 48 h, intracellular ROS level of RAW 264.7 cells raised 18.5 times as against untreated cells. Thereafter, RAW 264.7 cells activated with 1000 ng/mL of LPS for 48 h was used for the evaluation of FR. Unfortunately, the expression of FR over activated RAW 264.7 cells was not directly measured in this study; however, according to previous research, increased expression of FR [74] or FR mRNA [75] was detected in activated RAW 264.7 cells. In addition, an indirect CLSM imaging study was conducted in this study to validate those previous results. As seen in Figure 6G, LPS-activated RAW 264.7 cells revealed significantly higher fluorescence intensity after incubation with FOL-DOX&CAT-L than that of DOX&CAT-L while untreated RAW 264.7 cells showed indistinctive fluorescence intensity after incubation with either FOL-DOX&CAT-L or DOX&CAT-L. We presumed that activation by LPS could increase the expression of folate receptor over the surface of RAW 264.7 cells and then enhance the folate receptor-mediated internalization between FOL-DOX&CAT-L and RAW 264.7 cells, which eventually led to the higher fluorescence intensity.

As for the intracellular uptake study, after incubation with untreated RAW 264.7 cells, all three liposomes revealed concentration-dependent and time-dependent cellular uptake and no significant difference was observed (Figure 6C–E). On the other hand, after incubation with activated RAW 264.7 cells, DOX-L and DOX&CAT-L still showed a similar cellular uptake. However, FOL-DOX&CAT-L exhibited a 1.3-fold enhanced cellular uptake (after 8 h incubation) and 1.6-fold enhanced cellular uptake (at 20 μg/mL of DOX) compared with that of FOL-DOX&CAT-L incubated with untreated RAW 264.7 cells, which could benefit from the folate receptor-ligand interaction effect (Figure 6D–F).

Additionally, Figure 6B showed the intracellular ROS level of activated RAW 264.7 cells after incubation with three liposomes for 8 h. Compared with DOX&CAT-L, both DOX&CAT-L and FOL-DOX&CAT-L could significantly decrease the intracellular ROS level (*P* < 0.01) due to the decomposition effect of CAT on H_2_O_2_. Not surprisingly, a lower ROS level of FOL-DOX&CAT-L was observed than that of DOX&CAT-L (*P* < 0.05), which could further demonstrate the dual functions of FOL-DOX&CAT-L that is, folate receptor-mediated uptake and H_2_O_2_-decomposition effect.

### 3.5. In Vitro Cytotoxicity Study

**Figure 7:**
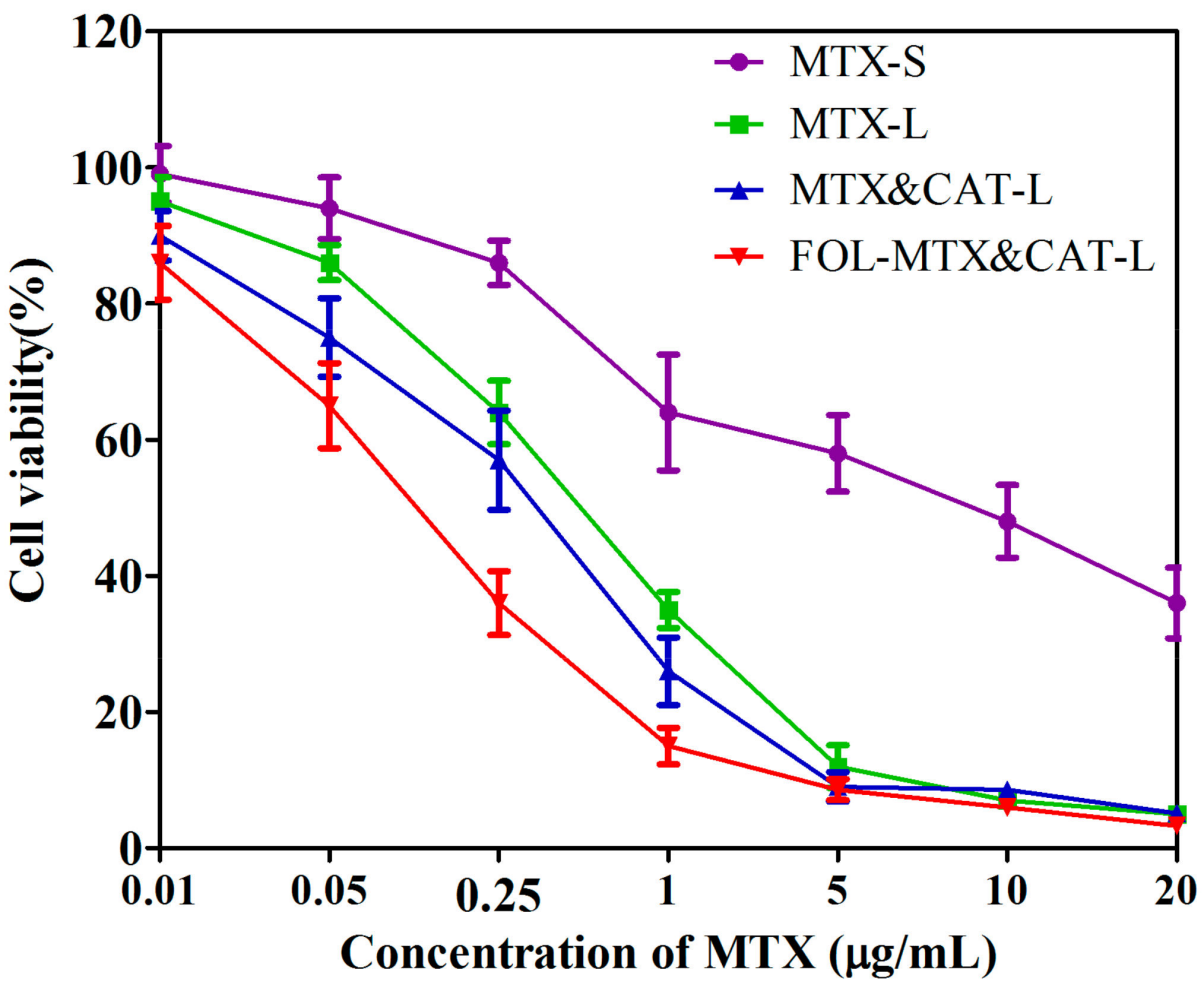
Cytotoxicity of TMX-S, MTX-L, MTX&CAT-L and FOL-MTX&CAT-L against activated RAW 264.7 cells as a function of MTX concentration after 24 h. Data are represented as Mean ± S.D. (*n* = 3).

Cytotoxic effects of three liposomes were investigated and compared to that of free MTX solution (MTX-S) in activated RAW 264.7 cells using MTT assay. As demonstrated in Figure 7, within the concentrations range 0.25–20 μg/mL, all three liposomes exhibited a stronger inhibitory effect on activated RAW 264.7 cells proliferation compared with MTX-S (*P* < 0.01). The IC_50_ of MTX-S was 7.128 μg/mL and the IC_50_ of MTX-L, MTX&CAT-L and FOL-MTX&CAT-L significantly decreased to 0.4775, 0.2746 and 0.1132 μg/mL, respectively. Moreover, within the concentrations range 0.05–1 μg/mL, FOL-MTX&CAT-L displayed a pronounced cytotoxic effect compared with MTX-L (*P* < 0.01) and MTX&CAT-L (*P* < 0.05). As mentioned above, FOL-MTX&CAT-L revealed accelerated drug release in the presence of H_2_O_2_ in vitro (Section 3.3) and FOL-DOX&CAT-L showed higher intracellular uptake (Section 3.4). Considering that the usage of a different model drug, for example, MTX and DOX, had no influence on the folate-targeting and ROS-responsive releasing feature of liposome, we presumed that FOL-MTX&CAT-L would display the similar intracellular uptake behavior with FOL-DOX&CAT-L. Accordingly, the highest cytotoxic effect of FOL-MTX&CAT-L could be benefit from its higher intracellular uptake and rapid drug release.

### 3.6. Pharmacokinetics and In Vivo Biodistribution

**Figure 8:**
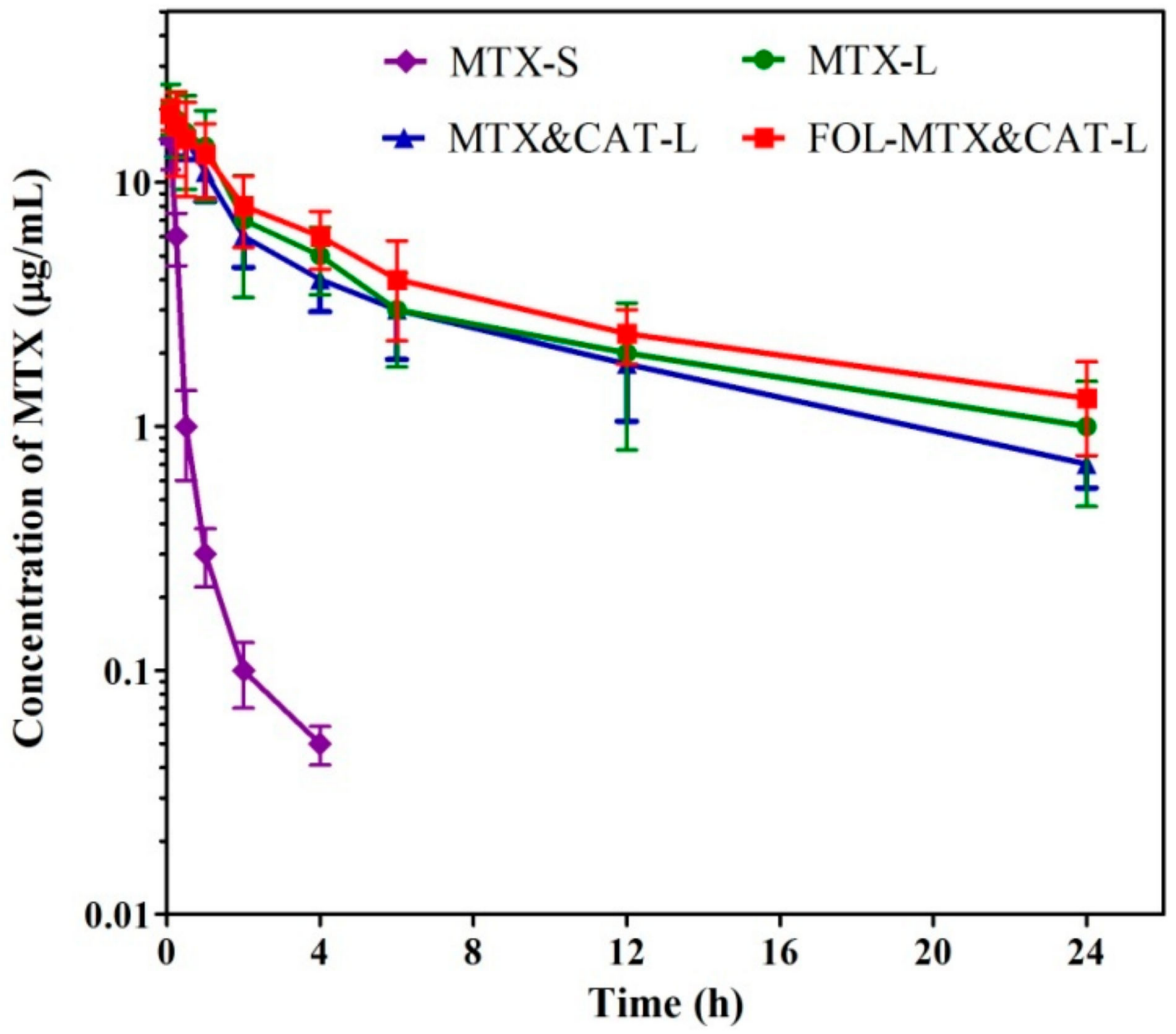
Concentration versus time curve of MTX in SD rats after intravenous administration of MTX-S, MTX-L, MTX&CAT-L and FOL-MTX&CAT-L. Date represents as Mean ± S.D. (*n* = 6).

MTX plasma concentration–time profiles were obtained after intravenous administration of MTX-S, MTX-L, MTX&CAT-L and FOL-MTX&CAT-L (equivalent dose of 4 mg/kg MTX) in male Sprague Dawley rats. Figure 8 showed that MTX plasma concentration declined rapidly following the intravenous administration of MTX-S and nearly undetectable 4 h later. However, the existence of PEG chains of three liposomes played a rather protective effect and significantly decreased the plasma clearance rate. Table 2 summarized the pharmacokinetic parameters of different groups. Compared to MTX-S, all three liposomes exhibited increased prolonged half-lives (T_1/2_) of 6.1, 7.4 and 7.6 times higher, respectively (*P* < 0.01) and mean residence times (MRT) of 6.5, 6.9 and 7.1 times higher, respectively (*P* < 0.01), which could result in greater accumulation of MTX in inflamed joints. The area under the concentration–time curve (AUC_0-24_), a major indicator of drug therapeutic efficacy, was improved by 16.1, 16.3 and 16.7 times for MTX-L, MTX&CAT-L and FOL-MTX&CAT-L, respectively (*P* < 0.01). Moreover, considering that no significant difference of pharmacokinetic parameters in three liposomes was observed, we supposed that the modification of folic acid and co-encapsulation of MTX and CAT had negligible impact on the pharmacokinetic behavior of liposomes.

**Figure 9:**
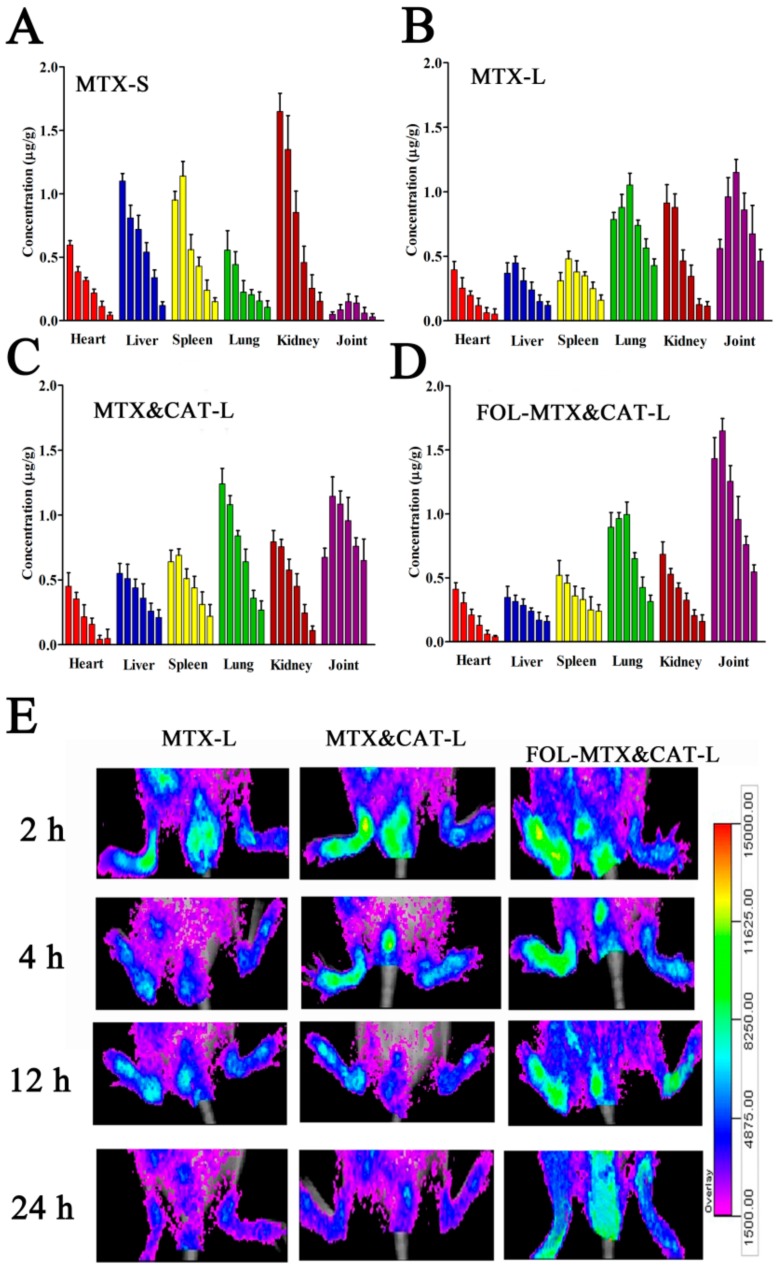
(**A**–**D**) Biodistribution profiles of MTX accumulation in the heart, liver, spleen, lung, kidney and joint of CIA mice following intravenous administration of MTX-S, MTX-L, MTX&CAT-L and FOL-MTX&CAT-L at a MTX dose of 4 mg/kg for 0.5, 2, 4, 8, 12 and 24 h (*n* = 3). (**E**) In vivo fluorescence imaging of CIA mice at 2, 4, 12 and 24 h after intravenous administration of DiR-loaded MTX-L, MTX&CAT-L and FOL-MTX&CAT-L by collecting images within the excitation wavelength of 720 nm and emission wavelength of 790 nm.

In vivo biodistribution of MTX-S, MTX-L, MTX&CAT-L and FOL-MTX&CAT-L after intravenous administration was evaluated by quantitatively determining the amounts of MTX in different organs and joints. As seen in Figure 9A–D, all three liposomes showed reductive accumulation of MTX in reticuloendothelial system (RES)-rich organs that is, liver and spleen when compared with MTX-S, indicating the PEGylation of liposomes could effectively reduce the uptake by RES system. In addition, after the administration of MTX solution, higher accumulation of MTX in kidney was observed, which could be responsible for its nephrotoxicity [76]. All three liposomes, however, revealed much lower amount of MTX in kidney and thus partially reduced its nephrotoxicity. Furthermore, all three liposomes exhibited higher MTX accumulation in the inflamed joints than the MTX solution (*P* < 0.01) and the AUC_0-24_ of MTX-L, MTX&CAT-L and FOL-MTX&CAT-L in the joints were 4.48-fold, 4.63-fold and 6.79-fold higher than that of MTX solution, respectively. The prolonged blood circulation time owing to PEGylation enabled the increased accumulation of three liposomes in joints through EPR effect. Accumulation of MTX in MTX-S and MTX-L reach the maximum level at 4 h post injection whereas that of in MTX&CAT-L and FOL-MTX&CAT-L reach the maximum level at 2 h post injection. Furthermore, compared with MTX-L and MTX&CAT-L, FOL-MTX&CAT-L displayed higher accumulation and AUC_0-24_ of MTX in the inflamed joints (*P* < 0.05). Several studies investigated the expression of folate receptors and ROS level from the perspective of RA animal models. Specifically, Chen et al. [77] established a LPS-induced mice arthritis model and injected a near-infrared fluorescence-labeled folate probe (NIR2-folate). Subsequent histological colocalization results indicated that NIR2-folate specifically colocalized with activated macrophages in the inflamed joints. Paulos et al. [78] developed the folate-linked haptens that could be targeted to activated macrophages. After treatment with this folate-hapten conjugate in CIA mice, activated macrophages with over-expression of folate receptors in the inflamed joints were eliminated and the symptoms of arthritis were ameliorated. In another research, Chen et al. [45] designed a nanoprobe for high sensitively sensing and bioimaging ROS level in CIA mice. Typically, compared to healthy mice, the CIA mice had significant enhancement of nanoprobe signal after injection for 12 h and the enhancement level of nanoprobe signal in the arthritic joint was about 5.1 times as compared to that of 2 h post-injection. Combined with these findings, we supposed that the highest accumulation of FOL-MTX&CAT-L in the inflamed joints of CIA mice could be associated with both folate receptor-mediated endocytosis and rapid intracellular drug release. Additionally, we also applied in vivo fluorescence imaging method to qualitatively observe and compare the distribution of three liposomes in joints (Figure 9E). As expected, FOL-MTX&CAT-L showed the highest fluorescence intensity at each time point, which was in accordance with the aforementioned results.

### 3.7. Therapeutic Efficacy and Safety

**Figure 10:**
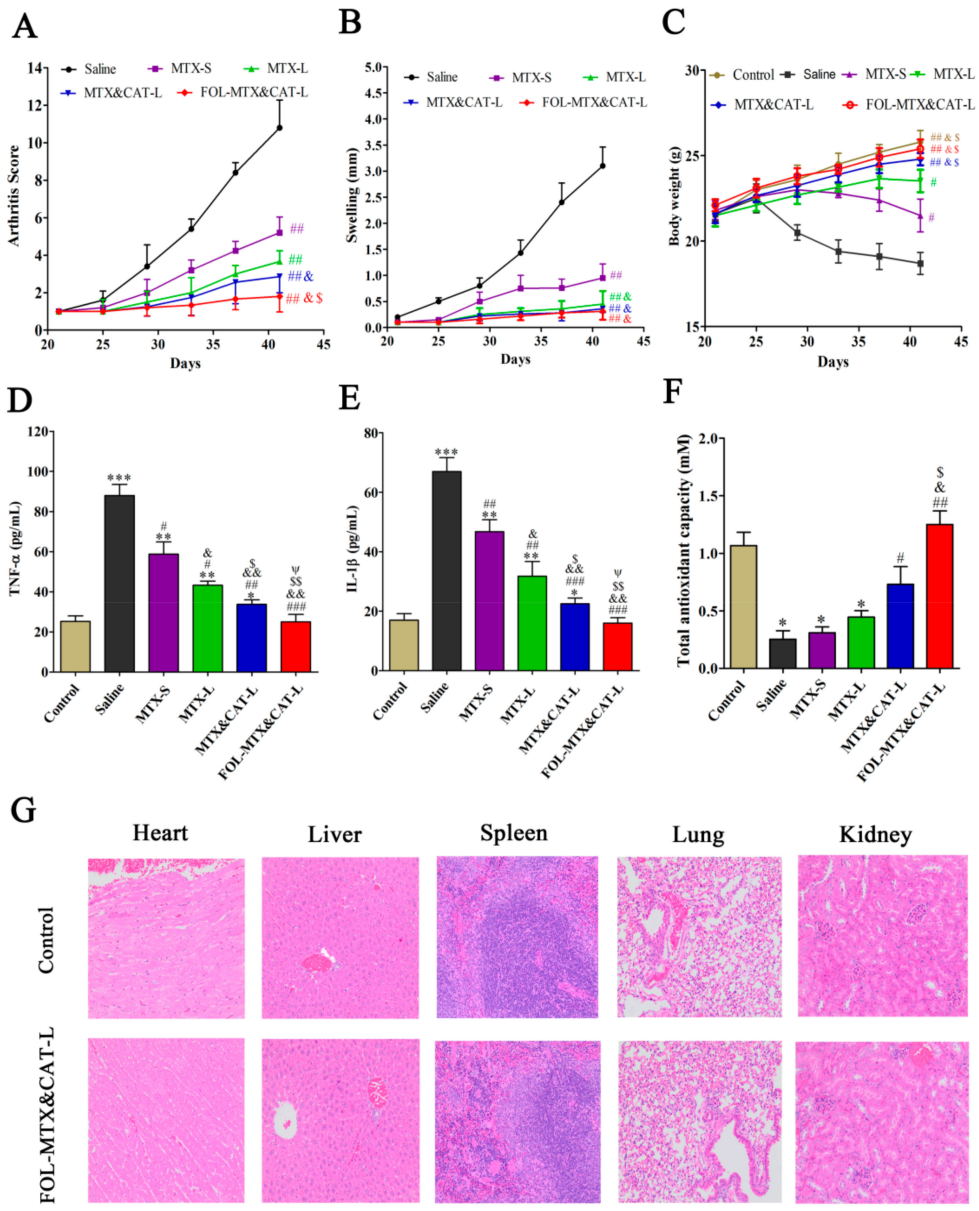
Therapeutic efficacy and safety results of collagen-induced arthritis (CIA) mice treated with saline, MTX-S, MTX-L, MTX&CAT-L and FOL-MTX&CAT-L. Healthy mice served as control group. (**A**) Arthritis score of mice in different groups. (**B**) Paw thickness (swelling) of mice in different groups. (**C**) Body weight of mice in different groups. Serum levels of (**D**) TNF-α, (**E**) IL-1β and (**F**) total antioxidant capacity of mice in different groups. (**G**) Histological results of heart, liver, spleen, lung and kidney of mice in control and FOL-MTX&CAT-L group by hematoxylin and eosin staining. Data are represented as Mean ± S.D. (*n* = 6). * *P* < 0.05, ** *P* < 0.01 and *** *P* < 0.001, vs. Control; ^#^ *P* < 0.05, ^##^ *P* < 0.01 and ^###^ *P* < 0.001 vs. Saline; ^&^ *P* < 0.05 and ^&&^ *P* < 0.01, vs. MTX-S; ^$^ *P* < 0.05 and ^$$^ *P* < 0.01, vs. MTX-L; ^Ψ^ *P* < 0.05, vs. MTX&CAT-L.

The therapeutic efficacy of MTX-solution and three liposomes were assessed in terms of arthritis index and paw thickness, which are crucial parameters to evaluate the anti-inflammatory effect. Figure 10A–B displayed the persistent increment of arthritis index and paw thickness in saline group indicating the successful modeling of CIA mice in this study. After the intravenous administration of MTX-S and three liposomes, arthritis index revealed a significant decline (*P* < 0.01) and the paw-swelling trend was notably reversed (*P* < 0.01) in all groups. Moreover, FOL-MTX&CAT-L group showed the lowest arthritis index while no significant difference of paw thickness between three liposomes was observed, which was consistent with previous literatures by using MTX-loaded liposomes [79,80].

Serum levels of pro-inflammatory cytokines TNF-α and IL-1β were measured as important markers or signs for the development of RA and to some extent, can be used as surrogate markers of therapeutic efficacy for antiarthritic drugs [81]. According to this criterion, elevated levels of both TNF-α and IL-1β in saline group reflected the severity of inflammation in CIA mice whereas remarkable reduction of both TNF-α and IL-1β in FOL-MTX&CAT-L group indicated the great therapeutic efficacy of FOL-MTX&CAT-L (Figure 10D–E). Duan et al. [80] prepared a calcium phosphate -liposome to deliver NF-κB-targeted small-interfering RNA (siRNA) and MTX for the treatment of RA. Similar decreased serum levels of TNF-α and IL-1β were measured. Additionally, we determined the serum level of total antioxidant capacity (TAC) in each group, which can reflect the global combined antioxidant capacity of all individual antioxidants such as manganese superoxide dismutase, catalase and glutathione peroxidase type 1 in serum [82]. Clinical study showed that the plasma TAC level was significantly lower in the RA patients than healthy patients (*P* < 0.05) [83]. Thus, the serum level of TAC could indicate whole free radical activity. As seen in Figure 10F, the serum level of TAC in saline group was significantly lower than control group (*P* < 0.05). After the administration of MTX solution and three liposomes, the results of TAC level in all groups showed a varying degree of increment. Notably, TAC level in FOL-MTX&CAT-L group was raised to be equivalent to the normal level.

Lastly, results of safety study showed that body weight in mice treated with FOL-MTX&CAT-L increased continuously from day 21 to day 41, similar to body weight in healthy, untreated mice (Figure 10C). Furthermore, H&E analysis of major organs and tissues in FOL-MTX&CAT-L and healthy group indicated that, compared with the control group, FOL-MTX&CAT-L had no apparent pathological toxicity on the major organs and tissues (Figure 10G).

## 4. Conclusions

In summary, we successfully designed a multifunctional liposome that is, FOL-MTX&CAT-L with folate receptor targeting and ROS-responsive drug releasing effects. The physicochemical properties and drug release of FOL-MTX&CAT-L were characterized and revealed excellent encapsulation efficiency, drug loading capacity and satisfactory ROS-responsive drug release. Cellular uptake and cytotoxicity of FOL-MTX&CAT-L in vitro exhibited improved uptake via folate-mediated endocytosis, resulting in a higher cytotoxic effect toward activated RAW264.7 cells. Moreover, in vivo enhanced pharmacokinetic behavior, increased accumulation of FOL-MTX&CAT-L in inflamed joints and reinforced therapeutic efficacy were observed. More importantly, FOL-MTX&CAT-L had a minimal toxicity toward major organs. The present findings demonstrated that FOL-MTX&CAT-L may be used as a potential drug delivery system for RA treatment.

## Figures and Tables

**Table 1:** Physicochemical property of MTX-L, MTX&CAT-L and FOL-MTX&CAT-L (*n* = 3).

Formulations	Particle Size (nm)	PDI ^a^	Zeta Potential (mV)	EE (%) ^b^		DL (%) ^c^	
				MTX	CAT	MTX	CAT
MTX-L	134.9 ± 2.6	0.17 ± 0.06	–8.5 ± 0.9	89.24 ± 3.18	—	7.18 ± 0.36	—
MTX&CAT-L	141.2 ± 3.2	0.18 ± 0.04	–8.8 ±1.1	84.65 ± 3.74	76.52 ± 2.62	6.75 ± 0.52	2.36 ± 0.42
FOL-MTX&CAT-L	145.5 ± 4.5	0.17 ± 0.03	–4.2 ± 0.8	86.72 ± 2.54	79.44 ± 1.85	6.94 ± 0.72	2.52 ± 0.26

^a^ PDI: Polydispersity index; ^b^ EE: Encapsulation efficiency; ^c^ DL: Drug loading.

**Table 2:** Pharmacokinetic parameters of different groups after i.v. administration (*n* = 6).

Parameters	MTX-S	MTX-L	MTX&CAT-L	FOL-MTX&CAT-L
AUC_0-24_ (h⋅μg/mL)	4.91 ± 0.08	78.86 ± 2.84 **	80.13 ± 4.52 **	82.22 ± 6.74 **
T_1/2_ (h)	1.23 ± 0.02	8.52 ± 0.82 **	9.16 ± 1.19 **	9.40 ± 2.06 **
MRT (h)	1.85 ± 0.06	11.96 ± 2.86 **	12.85 ± 3.63 **	13.08 ± 2.65 **

Data represented as Mean ± S.D. (*n* = 6). ** *P* < 0.01 vs. MTX solution.
